# Computer vision enables taxon-specific identification of African carnivore tooth marks on bone

**DOI:** 10.1038/s41598-024-57015-z

**Published:** 2024-03-22

**Authors:** Manuel Domínguez-Rodrigo, Marcos Pizarro-Monzo, Gabriel Cifuentes-Alcobendas, Marina Vegara-Riquelme, Blanca Jiménez-García, Enrique Baquedano

**Affiliations:** 1 Institute of Evolution in Africa (IDEA), Alcalá University, Covarrubias 36, 28010 Madrid, Spain; 2 Area of Prehistory (Department History and Philosophy), University of Alcalá, 28801 Alcalá de Henares, Spain; 3 Department of Anthropology, Rice University, 6100 Main St., Houston, TX 77005-1827 USA; 4 Institut Català de Paleoecologia Humana i Evolució Social (IPHES-CERCA), Zona Educacional 4, Campus Sescelades URV (Edifici W3), 43007 Tarragona, Spain; 5 Departament d’Història i Història de l’Art, Universitat Rovira i Virgili (URV), Avinguda de Catalunya 35, 43002 Tarragona, Spain; 6 Regional Paleontological and Archaeological Museum of Madrid, Plaza de las Bernardas S/N, Alcalá de Henares, Spain

**Keywords:** Archaeology, Anthropology

## Abstract

Taphonomic works aim at discovering how paleontological and archaeofaunal assemblages were formed. They also aim at determining how hominin fossils were preserved or destroyed. Hominins and other mammal carnivores have been co-evolving, at least during the past two million years, and their potential interactions determined the evolution of human behavior. In order to understand all this, taxon-specific carnivore agency must be effectively identified in the fossil record. Until now, taphonomists have been able to determine, to some degree, hominin and carnivore inputs in site formation, and their interactions in the modification of part of those assemblages. However, the inability to determine agency more specifically has hampered the development of taphonomic research, whose methods are virtually identical to those used several decades ago (lagged by a high degree of subjectivity). A call for more objective and agent-specific methods would be a major contribution to the advancement of taphonomic research. Here, we present one of these advances. The use of computer vision (CV) on a large data set of images of tooth marks has enabled the objective discrimination of taxon-specific carnivore agency up to 88% of the testing sample. We highlight the significance of this method in an interdisciplinary interplay between traditional taphonomic-paleontological analysis and artificial intelligence-based computer science. The new questions that can be addressed with this will certainly bring important changes to several ideas on important aspects of the human evolutionary process.

## Introduction

The identification of taxon-specific carnivore agency has become fundamental for the archaeological reconstructions of hominin and carnivore interactions. For example, at the behavioral level, it has been argued that hominin opportunistic behaviors may have been conditioned by their ability to parasitically extract resources from felid kills^1–3^. With the currently untestable interpretation of hominin interaction with extinct sabertooth felids^4^, for lack of modern counterparts, such assertions strongly rely on the identification of primary access to medium-sized carcasses by lions^1^ and to small-sized carcasses by leopards^5^ from bones accumulated at early sites. The alternative hypothesis (hominins were primary agents in carcass exploitation followed by durophagous carnivores) requires the identification of hyenas and other smaller durophagous carnivores on carcasses exploited by hominins at early archaeofaunal assemblages^6–9^. The major consequences in the interpretation of agency through the carnivore taphonomic signatures on fossil archaeofaunas underscores that the mere term “carnivore” is insufficient to address these questions^10,11^. Carnivore tooth mark frequencies are of limited value, since a multi-patterned lion-hominin-hyenid scenario would yield low frequencies of marks on long bone mid-shafts^12^, undifferentiated from a hominin-hyenid scenario^3,12,13^. Therefore, taxon-specific and carnivore-type determinations are essential to overcome the partial impasse driven by the frequencies and anatomical distribution of tooth marks. Although, in general, these traditional taphonomic methods could be used to diligently separate different carnivore agents, enough overlap exists in several instances to securely identify agency, due to equifinal processes and other preservation processes that affect the disappearance of tooth marks and skeletal parts^14^. The term “taxon-specific” is adopted here in a broad sense, referring not only to specific taxa (e.g., *Panthera leo* vs. *Panthera pardus*), but to specific carnivore types (e.g., hyaenids vs felids).

An additional advantage in the possibility of identifying taxon-specific carnivore agency affects hominin evolutionary history. From a human evolutionary perspective, it has been argued that more than half of our evolutionary trajectory was dominated by hominins being prey; namely, the australopithecine phase of hominin evolution^15,16^. The hominin fossil record is dominated by conspicuous evidence of carnivore impact, probably accounting for the paucity of complete or partial hominin carcass remains. Crocodiles have also been interpreted as predators of hominins^17^. Their taphonomic signature has been detected on *Homo habilis* paratype fossils^18,19^. Felid agency^20^, as well as hyenid agency^10,21^ have been interpreted from modifications found on hominin remains. This is of extreme relevance to detect the “shift in the balance of power”^22^, in which humans theoretically switched from prey to hunters^23^. Determining if (a) this process of hominin as prey ever existed, and (b) when and which hominins were affected by this trophic switch are major questions in human evolution. None of this will ever be known without being able to identify taxon-specific taphonomic signatures.

Taxon-specific experimental determination of tooth marks identified on bones has been already successfully attempted using geometric morphometric approaches^24–26^; however, these methods have been based on absolutely small sample sizes lacking sufficient statistical power. Although these models yield high accuracy, the agent-specific subsamples are insufficient to sample the range of tooth mark sizes and allometries for each agent, since probably larger marks are selected over smaller ones, because they are easier to model bi- and tri-dimensionally (see more in Discussion). The results, though, are really encouraging and are worth pursuing with larger experimental samples and proper sampling of agent-specific variance.

Computer vision analyses of tooth marks from larger experimental samples have also been successful at discriminating carnivore agency^10,11,27^. Computer vision uses standardized bidimensional images of tooth marks (pits and scores). In the only study where multiple carnivores were simultaneously compared, accuracy in discriminating five different types of agents reached 56% of correct identification^10^. This moderate accuracy resulted from the underperformance of the models because of a redundancy in the standardization of the images during image augmentation. When corrected, accuracy was > 70%. Here, we will use this method to provide a second generation of models that improve accuracy substantially using a larger and improved sample of tooth marks. Given the relevance of lions, leopards, hyenas and crocodiles as potential sources of scavengeable resources by hominins in African evolutionary scenarios, and also as potential hominin predators, we will use modern experimentally-derived tooth marks from these agents to generate a graphic library, and a series of models that can be used to objectively classify carnivore tooth marks on archaeofaunal assemblages and on hominin bones.

This study constitutes the methodological paper for forthcoming works applying this referential database to specific problems in the fossil record.

## Sample and methods

### Samples

**Figure 1:**
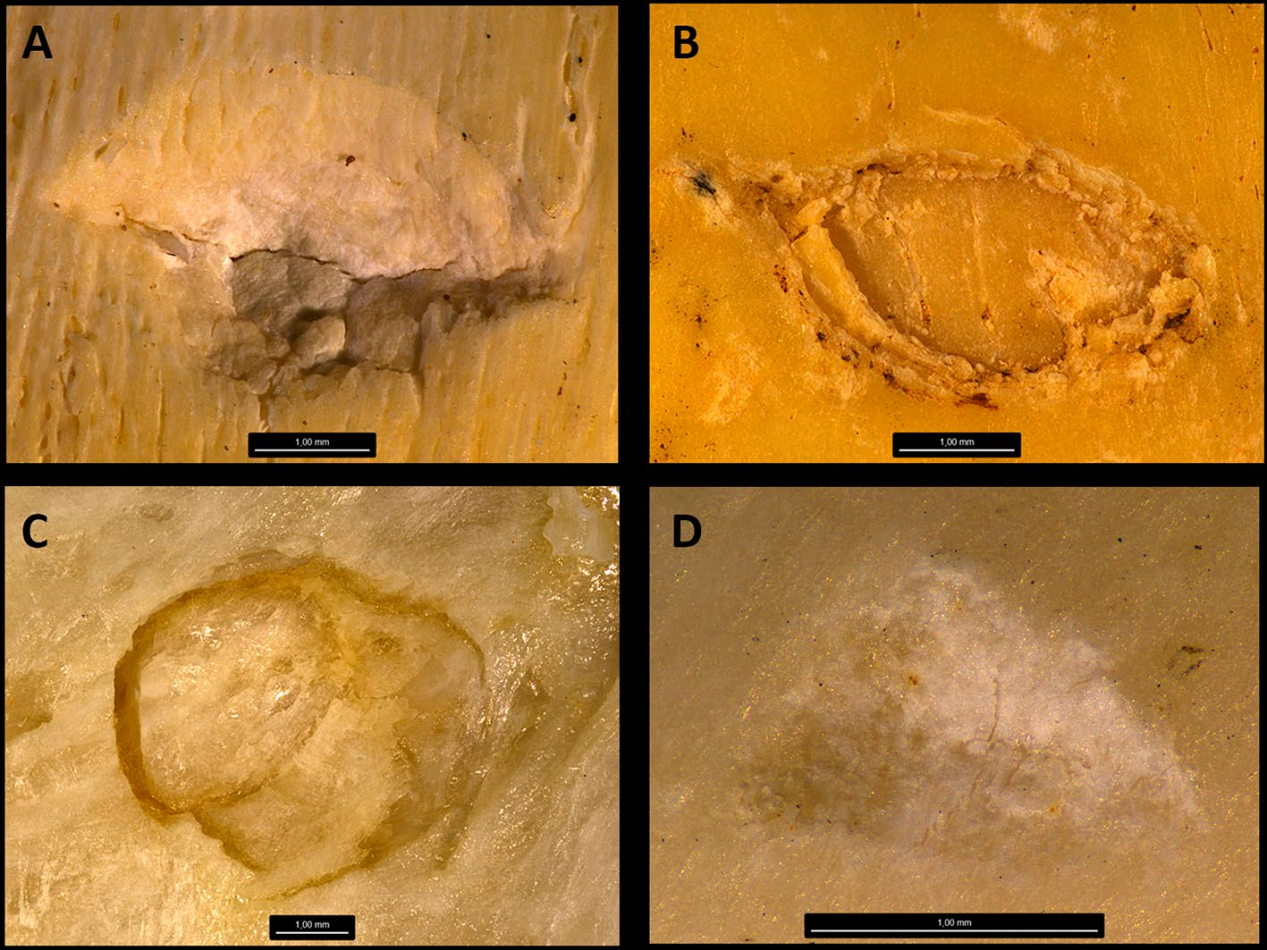
Examples of tooth pits from the four types of carnivores recorded. (**A**) Crocodile tooth pit. (**B**) Hyena tooth pit. (**C**) Lion tooth pit. (**D**) Leopard tooth pit.

**Figure 2:**
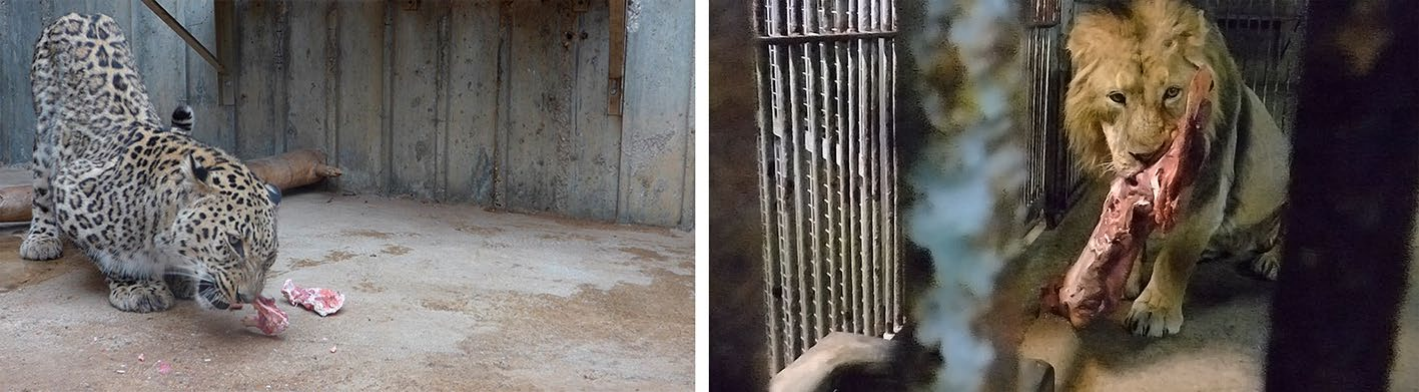
Examples of feeding episodes for leopard (left) and lion (right) from the Madrid Zoo.

A total of 1256 tooth marks (including tooth pits and scores) were used for the analysis (Fig. 1). This experimental work was aimed at providing a solid reference image database for the four most common extant carnivores interpreted as potentially interacting with hominins in the African past: lions, leopards, hyenas and crocodiles. The tooth mark samples are divided as follows: lions (n = 264), leopards (n = 544), hyenas (n = 364) and crocodiles (n = 84) (Fig. 2). Previous analyses of bidimensional tooth marks were carried out using a Optika binocular microscope^10^, whose depth of field was not efficient. This created unfocused areas in several of the photographed marks. In order to improve image quality, a new image bank was created using a Leica Emspira 3 digital microscope, which is capable of capturing not only bidimensional, but also tridimensional images. This microscope can stack overlapping images of the same mark removing any unfocused area. Here, and in contrast with previous studies, we used color photographs, using mostly marks under variable magnification for pits (ranging from x7 to x60 depending on mark size) and systematically under x30 magnification for scores. Also in contrast with previous analyses, we decided to lump together tooth scores and pits, instead of analyzing them separately. This removes any ambiguity in the identification of pits and scores and their potential overlaping boundaries. It also increases the modeling power, since each class is learnt by each algorithm through a bigger dataset.

Most of the experimental sample was also new. Carcasses from small (goat, sheep, boar, pig), medium-sized (deer) and large animals (cows) were used in the experiments, as described below.

#### Experimental series with hyenas

Four spotted hyenas (*Crocuta crocuta*) were used; all of them from the private Madrid Safari Reserve (Spain). These included an adult (16 years old) female weighing 30 kg, an adult (18 years old) male weighing 35 kg, an eight year-old adult female weighing 32 kg, and a 6-year-old adult female weighing 28 kg. The diet of all of them usually consists of 3 kg of lean meat with bone two days a week, and fruit the other days. For this experiment, a collection of disarticulated and defleshed bones of adult deer (*Cervus elaphus*) was used, following all sanitary protocols. Thus, a total of 27 femurs, 6 humeri, 9 radii and 14 tibias, all of them from both sides, were used. All the hyena individuals consumed the remains individually, removing them from their resting enclosures shortly after their initial consumption, with the aim of recovering the greatest number of remains possible. The adult male consumed 6 tibias, 1 humerus and 7 femurs; the adult female consumed 9 femurs, 2 humeri, 3 radii and 1 tibia; and the other adult female modified 4 femurs, 2 humeri, 2 radii and 1 tibia. Bones were collected after 1 h of exposure, before they were fully broken and deleted by hyenas, and then cleaned with a solution of neutral detergent and boiling water.

Tooth marks were also collected from the Eyasi spotted hyena den^28^. This assemblage is anomalous because it is a breeding lair where most of the fauna was composed of ovicaprids and damage was mostly done by hyena cubs. The intention for doing this was to broaden the range of tooth mark sizes and shapes, since the unworn dentition of subadult individuals resulted in sharper marks, compared to adults.

#### Experimental series with lions

Three adult Asiatic lions (*Panthera leo persica*) were used to carry out this experiment, all of them from the Madrid Zoo (Spain), where they live in captivity (Fig. 2). Specifically, these were a 12-year-old male weighing 198 kg, and two 8-year-old females weighing between 150 and 160 kg. During the experimental series, the carcasses provided were adjusted to the usual diet of the lions, consisting of 6 kg of lean meat for the male, and 6 kg between the two females for six consecutive days, and 1 day of fasting. Thus, a total of 16 bovine (*Bos taurus*) slightly defleshed limbs were used, distributed as follows: 8 front legs (scapula, humerus, radius-ulna, and carpals) and 4 hind legs (femur, patella, tibia, calcaneus, talus, and tarsals). All the anatomical elements were in anatomical connection, being fed to the carnivores separated by sex. These were introduced into the animals’ resting area at 8 pm and collected at 8 am the following morning by the zookeepers, who photographed and videotaped their consumption. Thus, the females consumed a total of 4 front limbs and 6 hind limbs, and the male consumed 4 front limbs and 2 hind limbs. This difference is because the females were provided with 2 limbs at each feeding episode, to avoid the rivalry and fighting.

An additional sample of lion tooth marks were used. This was obtained from experiments conducted with captive lions from the private Cabárceno reserve (Cantabria, Spain) reported in a previous work^10^, and the original images obtained thereof were also included. There, animals live in open areas comprising several thousands of square meters, which impacts their behavior since they do not display the usual stereotypic behaviors associated with captivity or with caged carnivores^29,30^. In this case, 11 adult lions from this reserve were given 60 limb bones from prime adult and older juvenile equid (*Equus ferus caballus*) individuals by the staff, following their usual feeding protocol. Limbs were detached from the trunk at the proximal humeral and femoral heads and then fed to the animals, discarding the metapodials. Bones were collected after 1 to 4 days of exposure, when they were completely defleshed and abandoned by the lions, and then cleaned with a solution of neutral detergent and boiling water for an average of 1.5 h and left to dry.

#### Experimental series with leopards

Three Persian leopards (*Panthera pardus saxicolor*) were used to carry out this experiment, all of them from the Madrid Zoo (Spain), like the lions described above (Fig. 2). In this case, the carnivores were two 4-year-old males weighing 60 kg, born in the Madrid Zoo itself, and an adult 9-year-old male weighing 70 kg. During the experimental series, the carcasses used, as in the case of the lions, were adjusted to their habitual diet. Thus, a total of 12 complete adult sheep limbs (*Ovis aries*) were fed to the leopards. All the limbs were supplied to the leopards in anatomical connection, separating the carnivores by age to avoid hierarchical disputes. Like the experimental series with lions, the experimental work was carried out by the zookeepers responsible for the animals, who carried out an exhaustive record of the process. Thus, the young leopards consumed a total of 4 front limbs and 4 hind limbs, and the adult leopard consumed 2 front limbs and 2 hind limbs.

Once the remains of each of the experimental phases were recovered, all of them were cleaned. This was done by boiling them in water for 6 h, and submerged in a solution of water and hydrogen peroxide for 24 h, with the aim of removing excess meat and fat remains, in order to enable the documentation of the modifications created.

An additional sample of leopard-impacted carcasses was also used. They were used in an experiment with a leopard held in the Bahari Zoo (Dar es Salaam, Tanzania). Over the course of several weeks, the leopard was fed 44 articulated, skin- and fleshed-covered fore- (humerus and radius-ulna only) and hindlimbs (femur and tibia only) of 11 goat (*Capra hircus*) carcasses. The leopard was confined to a 2.5 m × 4.5 m cage for the entirety of the study. Limbs were presented to the leopard at 9:00 am and all feeding residues were collected at noon. This experiment was already reported earlier for an analysis of carcass portion deletion by leopards^29^. Tooth marks documented in this experimental sample were observed and photographed using an Optika binocular microscope and a 3 Mpx digital camera (OptiCam3). In order to overcome problems with the depth of field described above, multiple images of the same mark were taken focusing on different parts of the image, and then processed with an image enhancing software. Pits were documented at variable magnifications to adjust the pit’s borders to the image frame, while scores were documented at a fixed magnification of × 30. The depth of field on that specific camera and microscope combo forced us to use focus stacking techniques in order to generate images without any blurry or out of focus areas. To do so, we took different images of each specific mark with variable focus, and then we merged them together using the native “Focus Stacking” command of Adobe Photoshop. This extra processing, while lengthy, ensured that these images would not produce any extra noise in the sample, and they could be used without cropping.

#### Experimental series with crocodiles

All the crocodiles used in the experiment were females: one small (1.3 m in length from nose to end of tail), two medium-sized (1.8 m) and five large individuals (2.3–3.10 m). They were fed in an enclosure area of the Faunia zoo (Madrid, Spain), once a week over 4 complete months with 19 partial carcasses. Carcasses were collected after 15 h of exposure to the crocodile, even though most part of the feeding took place during the first hour. The feeding process was monitored for the first 1.5 h, to be able to relate each carcass to specific individuals. Monitoring by the researchers stopped after carcass remains were abandoned by crocodiles. Zookeepers, then, kept on monitoring crocodiles at regular intervals to document if carcass remains were further accessed by crocodiles. All feeding episodes were captured through photographs and videos. These carcasses were composed of articulated limbs of suids (pig and boar) and bovids (sheep and cow): four forelimbs and five hindlimbs of suid, six sheep forelimbs and three hindlimbs of cow. In all cases, sheep limbs were articulated to their respective scapulae. For the suid carcass sample, only in one case was the scapula attached to the limb. Four pelves were articulated to some of the suid hindlimbs and two pelves were articulated to two cow hindlimbs.

Bone cleaning was done by boiling carcass remains in water for a few hours, and then subsequently submerged in a solution of water and hydrogen peroxide for 24 h.

Given that the focus of the present study is its posterior application as a referential dataframe for analysis of bone carnivoran tooth marks on fossils bones in Africa, we did not add any canid to the dataset, given that we did not have access to wild dog (Lycaon pictus) modified-bones, but only wolf-modified bones from our experiments with carnivores in the Iberian peninsula.

### Deep learning methods

Here, we used a deep learning (DL) computer vision approach, based on the use of bidimensional images of tooth marks obtained with magnification, in order to capture all the microscopic features inside the grooves and their boundaries. The deep convolutional neural network format was drawn from transfer learning (TL) (i.e., pre-trained architectures). We used TL because of the prior training on millions of images that pre-trained the network on a large diversity of morphological items, thus enhancing its capability to better capture the intricacies of the microscopic features in bone surface modifications (BSM). We had tested these TL architectures against similar models using the same raw convolutional neural networks and the performance of the pre-trained network was superior to the untrained network. Given the success of a set of models with other taphonomic analyses of BSM^10,27,31–33^, we used the same sequential and residual architectures for the present study; namely, ResNet 50 (version 1.0)^34^, VGG19^35^, Densenet 201^36^, and EfficientNet B7^37^. We generated individual models and, subsequently, adopted an ensemble learning (EL) approach. The EL method used here consisted of using the four models as base learners, and then a stacking process was implemented, by using a random forest and an extra-gradient boosted tree as the meta-learner. The number of estimators used in hyperparameter tuning was 100.

Prior to the DL analysis, the original 1256 tooth mark images were divided into a training set (75% = 942 images) and a testing set (25% = 314 images). This training/testing split is the customary protocol for machine learning models. To randomize the process of training/testing selection, all images were randomly allocated to both sets. In addition to the original large sample size, all the architectures were used with image augmentation to improve their training, since this technique has been shown to reduce the chances of overfitting^38^. The training image data set was augmented through the following procedures: random shifting of width and height (20%), modification of shear and zoom range (20%), horizontal flipping, and a rotation range of 40°. Image standardization, using bidimensional matrices for standardization and centering, was carried out using each architecture’s preprocessing functions. All images were reshaped to 250 × 200 pixels. The selection of this size was random, but we included a high number of pixels aiming at a better discrimination by the algorithms of all the microscopic features and nuances of each tooth mark. Given that most tooth marks seem very similar to the human trained eye, this high definition could help the DL process to find the classificatory differences. The DCNN models were elaborated using the Keras (2.4.3) Application Programming Interface (API) with a Tensorflow (2.3.0) backend. Computation was carried out on a GPU HP Z6 Workstation using a CUDA computing (cuDNN) environment. All code was made using Python 3.7.

Prior to the selection of activation function and optimizer, an exploratory use of TL models was performed with different combinations, as we recommended in prior experiments^39^. For the exploratory phase, we used two activation functions (ReLu and Swish) and three optimizers (Stochastic Gradient Descent [SGD], Adam and Adagrad). For the image data set at hand, the best combination resulted using the “relu” function and the SGD optimizer (with a learning rate of 0.001 and a momentum of 0.9). Therefore, for each of the TL models used, we used the best performing combination of activation function and optimizer. The last fully connected layer of the network used a “softmax” activation. The loss function selected was categorical cross-entropy^38^. Accuracy was the metric selected for the evaluation of the classification process. F1 score values were also obtained to assess balanced accuracy, given the imbalanced nature of the original dataset. Training was made using mini-batch kernels of size 32. Testing was made using mini-batch kernels of size 20. Weight update was made using a backpropagation process of 100 epochs.

Training graphs for accuracy and loss were also used in order to assess over- and underfitting training processes. The TL architectures implemented regularization methods based on Dropout^40^. Dropout consists of the random dropping (i.e., ignoring) of selected neurons during training. This results in DCNN networks that are less reactive to specific neuron weights, producing a network that is more adapted to implement better generalization and less likely to overfit from the training data. For the present study, we adopted a Dropout rate of 30%. All the images and code are accessible in a public repository: 10.7910/DVN/MLDCIC.

## Results

The four DL architectures (Resnet50, EfficientNet B7, VGG19 and Densenet 201) yielded accuracy estimates > 80% of the testing data set. The most successful model was a Resnet50 architecture using a SGD as the optimizer and reLu as the activation function, which yielded 88% of correct identification of the testing 314 images of tooth marks from the four carnivores (Table 1). Resnet50 was followed by Densenet 201 (84.3% of accuracy), and VGG19 and EfficientNet B7 (both with 80% of accuracy) (Table 1). The average accuracy for all models is 83%; something that is replicated with the ensemble analysis of all the models together, when using an extra randomized boosted tree algorithm (accuracy = 81.9%) or a random forest (accuracy = 82.5) as the metalearner of a stacked EL model.

**Table 1:** Accuracy and loss information of each of the four models.

Model	optimizer	Activation function	Accuracy	Loss
Resnet50	SGD	Relu	0.88	0.407
VGG19	Adagrad	Relu	0.80	0.541
EfficientNetB7	Adagrad	Swish	0.80	0.501
Densenet 201	SGD	Relu	0.843	0.409

The combination of optimizer and activation function for each model is the one showing the highest accuracy.

**Figure 3:**
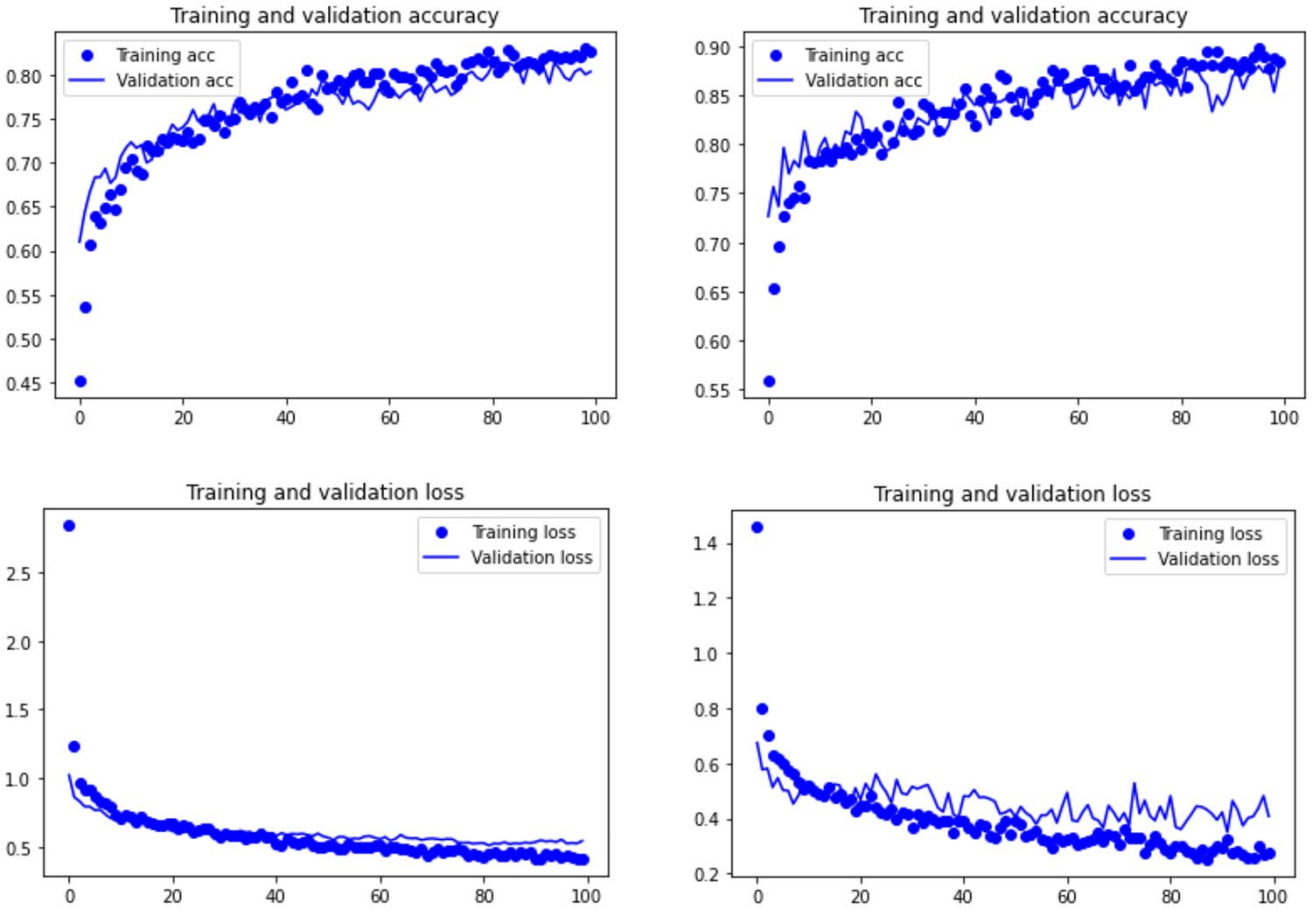
Model accuracy (upper) and loss (lower) for VGG19 (left), and Resnet50 (right).

**Figure 4:**
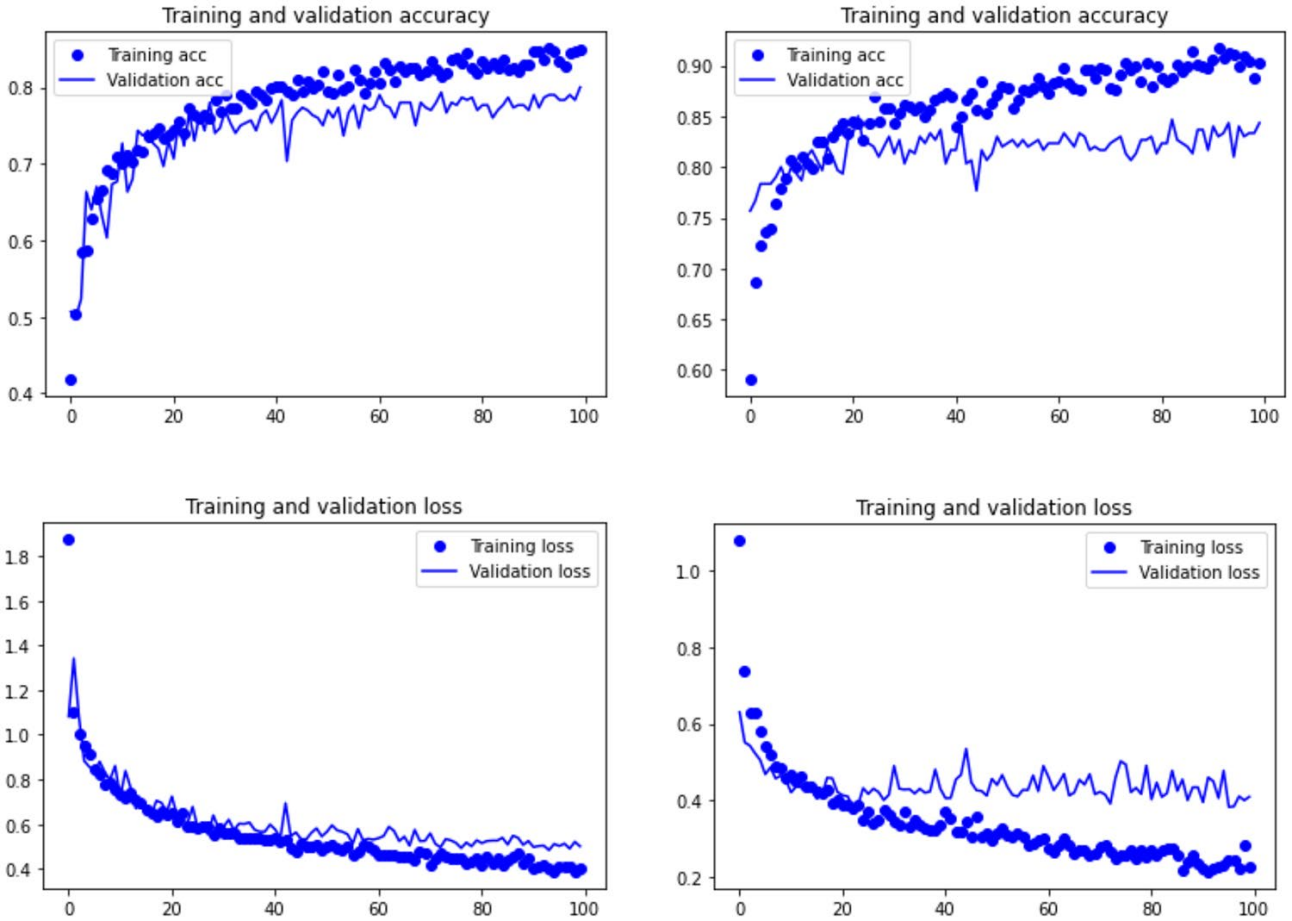
Model accuracy (upper) and loss (lower) for EfficientNetB7 (left), and Densenet 201 (right).

When observing the learning graphs, it can be seen that the dropout regularization method has prevented overfitting in most models. The best model (Resnet50) shows not only the highest accuracy, but also the best training fit (Fig. 3). Training was smooth, and both the training and validation sets progressed equally without any trace of overfitting. This was also documented on the slightly less accuracy VGG19 model, where the fit between the training and testing sets is even better (Fig. 3). In contrast, the learning process in the EfficientNet B7 and Densenet 201 models shows some stagnation after epoch 40, because of an increasing overfitting of the training set (Fig. 4). Despite this, both models stagnated with 80% and 84% of accuracy. This means that although they were unable to increase their knowledge, they still performed fairly well in classifying the testing sets.

**Figure 5:**
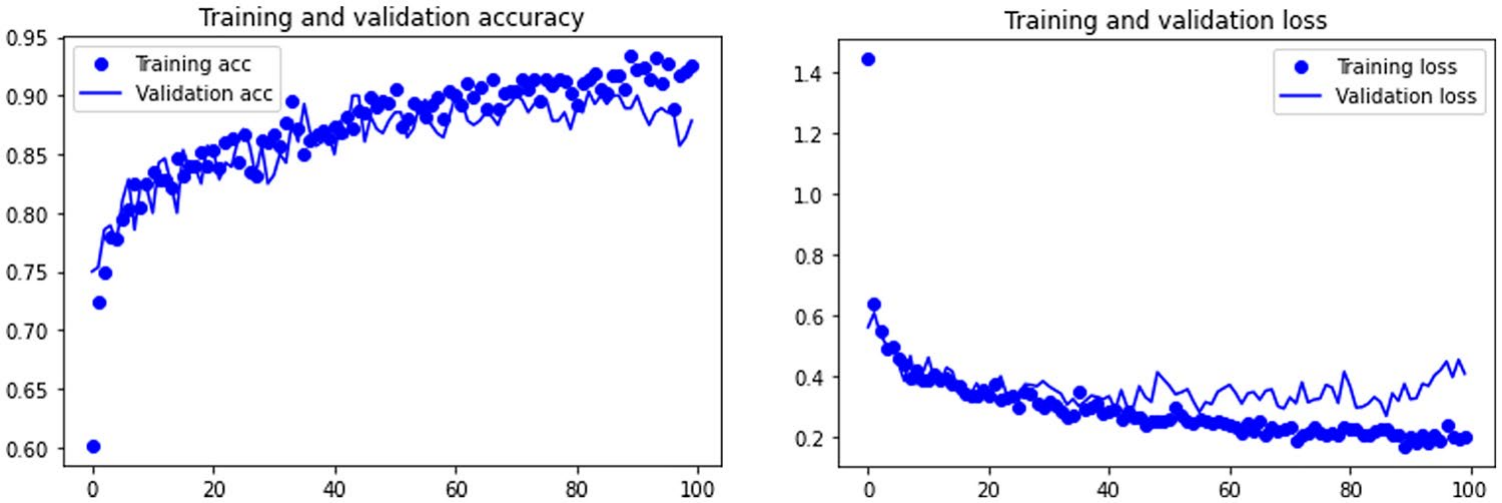
Model accuracy (left) and loss (right) for Resnet50 applied to the three-carnivore sample, excluding crocodiles.

In all the models, the classification error is very low for hyenas, lions and leopards, and most of the misclassification is documented in crocodiles (Supplementary Information). For example, the F-1 score values for the most accurate model (Resnet50) shows: hyena (f1 = 0.88), leopard (f1 = 0.90), lion (f1 = 0.87), crocodile (f1 = 0.60). The pattern is the same in the second most accurate model (Densenet 201): hyena (f1 = 0.84), leopard (f1 = 0.89), lion (f1 = 0.80), crocodile (f1 = 0.59). The reason could be the significantly smaller sample size for crocodiles. Given the higher F-1 score values for the other three agents for which there is a larger sample size, we predicted an equally high resolution if using only those three carnivores, discarding the smaller crocodile sample, which is making the global sample fairly unbalanced. When running the most successful model (Resnet50) on those three samples, it yielded exactly the same accuracy of 88% (loss = 0.408) as the original four-sample model, with the following F1 scores: lion (0.86), leopard (0.88), hyena (0.87). Slight differences with the previous result are explained by the randomness in the loss function, backpropagation and weight estimation. This shows the minimal impact of the imbalanced sample in the classification of all agents caused by the inclusion of the crocodile tooth marks (Fig. 5).

## Discussion

Previous methods attempting to differentiate among carnivore taxa were focused on the bidimensional metrics of tooth pits and scores. The diversity of body sizes and dentition among mammalian carnivores prompted investigations into the potential utility of tooth mark sizes for distinguishing carnivore species^41–43^. Selvaggio and Wilder demonstrated the feasibility of differentiating functionally distinct carnivore types (e.g., some felids from hyenids) by analyzing tooth mark shape (ratio of major axis to minor axis) and size (area in millimeters) on cancellous, thinning cortical, and dense cortical bone. Posterior experimental findings by Fernández-Jalvo and Andrews^44^ further supported the differentiation of carnivoran groups, based on their study of carnivore tooth marks, noting variations in tooth pit sizes depending on the bone element. Despite potential confounding factors, they categorized carnivore damage into three types based on mark size: small (< 1 mm), mixed (1–4 mm, indicative of small or large carnivores), and larger carnivores (> 4 mm). Dominguez-Rodrigo and Piqueras^42^ argued for the use of tooth pit sizes to distinguish between small and large carnivores, although they acknowledged ambiguity in differentiating specific carnivore taxa.

Experimental data from Delaney-Rivera et al.^43^ demonstrated a significant overlap in tooth pit dimensions across carnivore size and taxon. While they broadly supported the three-group scheme proposed by Dominguez-Rodrigo and Piqueras^42^, the observed overlap cautioned against relying solely on tooth pit sizes to determine carnivore type or specific carnivore taxa. Nonetheless, Delaney-Rivera et al.^43^ differentiated three carnivore types based on diaphyseal tooth pits: < 2 mm for small carnivores and medium-sized felids, 2–4 mm for various medium and larger carnivores, and > 4 mm for larger carnivores such as hyenas, lions, and large dogs.

These observations were further supported by work by Andrés et al.^45^, who investigated dimensional differences in tooth marks (pits and scores) on bones altered by small, medium-sized and large carnivores. In their study, Andrés et al. noted a significant overlap in tooth pit dimensions irrespective of carnivore size and taxon. While these authors confirmed some differentiation among carnivore groups based on tooth pit sizes, caution was advised due to observed overlap. The study highlighted challenges in reliably attributing single marks to specific carnivore groups, particularly those < 4 mm in size. Similar conclusions were reached in the dimensional study of tooth marks by Fourvel^7,46^. These conclusions have received recent support from the study of canine tooth marks (punctures) generated by different types of carnivores^47^. Experimentation with carnivores had traditionally paid special attention to the bulk of tooth marks on bones, mostly generated through the action to the tooth cusps of premolars and molar teeth. Canine-inflicted tooth marks also display the same ambiguity spectrum when targeting the taxonomic differentiation of carnivore taxa. Although tooth punctures have the advantage of reflecting not only tooth size, but also, depth of crown penetration into bone, Brugal and Fourvel^47^ could only determine dimensional boundaries for small, medium-to large and large carnivora, with no taxonomic resolution, and stressing that small punctures could be the result of carnivores of any size.

In summary, all these studies suggest some degree of correlation between carnivore size and tooth mark sizes beyond certain thresholds. Small carnivores predominantly leave small tooth marks, while larger carnivores are capable of inflicting both small and larger marks. However, attributing single marks to specific carnivore groups remains challenging, particularly for marks < 4 mm, which cannot be reliably used to distinguish among carnivore types. This is unfortunate because such marks make up most of the tooth marks in any given carnivore-modified bone assemblage.

Fortunately, geometric-morphometric (GMM) analyses of tooth marks have shown a higher discriminatory power^24–26^; however, until they are based on statistically meaningful samples that incorporate the full range of tooth mark forms within each carnivoran taxon, their reliability is questionable. Such published studies using GMM methods are probably biased by mark selection, since we know that several of the experimental assemblages used contain far more tooth marks than those selected^30^. If selection is biased towards the larger marks, this creates uncertainty as to whether allometric-variation that could be agent-specific is effectively captured by those analyses.

In some studies, one-class sample was as low as 21 marks, and the highest one was 34 marks^48^. In other studies, sample size is even smaller, with as few as 20 marks per mammal carnivore, and only 9 crocodile marks^49^. Such small sample sizes cannot capture within-agent variance, and therefore, classification reliability has the potential of being substantially biased. To date, the largest sample comparing multiple carnivores using GMM methods does not include any sample with > 90 marks per agent, and ranges from a maximum of 89 to a minimum of 53 tooth marks per class^50^. This is prior to excluding from 3 to 10 marks per agent, because they introduced a greater degree of variance (i.e., more potential inter-agent overlap). Although high accuracy estimates are obtained when using these small samples through machine learning algorithms, these may not be reliable. Reliability consists of high power (i.e., high confidence) in classification. Such models should reflect not only high accuracy, but also clear separation of samples in shape and form spaces when plotted in Euclidean spaces. Some of these studies are reported with principal components scatter plots without 95% confidence intervals, but intense overlap of data in point clouds^25,51^. Other studies report such confidence intervals, but show intense overlap of tooth marks from different carnivores in otherwise-highly accurate models^48,52,53^. Such a contradiction can be understood by the way classical discriminant methods (i.e., LDA [linear discriminant analysis]) and machine learning algorithms work. Item classification results from the highest probability out of the different options, even when such a probability has low statistical power. For example, in a three-class problem, an item is classified as class A if it has a minimum of 34% of probability (with the remainder [33.3%] equally distributed among the alternative classes), even if there is a 66% chance of error. This results in high accuracy but low reliability. See Table 3 in Ref.^53^ as an example. Even when methodological sophistication is displayed^54^, the ultimate fact is that intense overlap of marks from different agents in Euclidean space precludes highly accurate models from being reliable because of their potential low power. Bone surface modifications from the archaeological record should be classified with higher confidence, and only high power models should be trusted. As an example, DL models produce probability per classified mark, and in the application of highly accurate models to the archaeological record, only those marks classified with high confidence (> 70% of probability) have been accepted as reliable^9^. Even when high probability might be obtained, this is only reliable as far as the models upon which it is based truly sample agent-specific variance. This does not mean that GMM approaches are inadequate for the study of carnivore tooth marks. We are confident that when these shortcomings can be overcome, GMM methods have the potential of being equally successful. Until then, all published interpretations should be taken with caution.

The DL models described in the present work provide a more accurate differentiation of tooth marks (pits and scores) produced by lions, leopards, hyenas and crocodiles than previously reported in other CV studies^10^. The models have learnt better by lumping tooth pits and scores together, instead of treating them as separate samples, probably because this created enlarged referential samples from which to train. An increase of the quality of each image documented through the Leica Emspira 3 microscope is also a contributing factor in the final accuracy values. In this precise image data set, the “relu” activation function seems to provide the best discrimination. Both Adagrad and SGD achieved a better performance than the alternative optimizer (i.e., Adam) depending on the model.

These results are encouraging because they enable an objective way of differentiating carnivore types when analyzing bone surface modifications, namely, tooth marks. This opens the door to the possibility of efficiently addressing agency in bone assemblage formation and modification. Given the better performance of these models than previous models, we see an improvement in the interpretation of agency in the early Pleistocene record where agency discrimination was attempted earlier. For example, in the analysis of felid and hyenid impact on the DS 22b early Pleistocene archaeofaunal assemblage, with the exception of three elements, it was clearly shown that the majority of carnivore damage on the hominin-accumulated assemblage was caused by the secondary action of hyenas^9^. Application of the new models and referential database should further confirm or modify this interpretation. In addition, new sites are being analyzed following these protocols, like the pene-contemporaneous sites of PTK and DS 22a, hoping to yield a signal on the specificity of carnivore agency in the modification on the taphonomically anthropogenic assemblages from Olduvai Bed I.

A complementary application of these methods can be effective in addressing paleoecological reconstructions of different carnivores and their impact at a paleolandscape scale. We are currently using this approach to determine which carnivores were more active in portions of the paleolandscape in the 1.7 million-year-old HWK-FLK West site complex at Olduvai Gorge (Tanzania). At a diachronic scale, it would be interesting to compare moments of more or less hominin activities on the landscape with those of other carnivores and determine which types of ecological interactions of community predominance (felid-hominin, hominin-hyenid) were more common at different times.

Likewise, the extreme relevance of felid agency in modeling the first half of the human evolutionary process, by depicting australopithecines as systematic prey of leopards (and other similarly-sized felids) can be for the first time tested following these models. The recent evaluation of modifications found on 27 australopithecine fossil remains suggest that it was hyenas, instead of felids, that were responsible for the documented bone damage^26^. This calls for other methods to reassess these interpretations. Should computer vision models using these visual libraries, or even more complete ones in the future, determine that there are no predominant felid taphotype signatures on australopithecine bones, that would cast a shadow on the traditional leopard-hominin model, and part of our evolutionary past should be reevaluated. In fact, this would allow for the first time to determine if (a) there was a passive prey stage in our evolution, and (b) when the shift in the balance of power took place, and with which adaptive and anatomical innovations. The present study and its referential image library will provide the founding stage for these types of analyses.

An additional impact of this methodological advance lies in its application to modern ecology. Studies aiming at assessing carnivore impact on different habitats within an ecosystem can extract indirect information of carnivore taxa and their impact on biocoenoses, by identifying their traces on the bones of their prey. This can help in assessing how abundant specific carnivore types are within each ecosystem.

## Conclusions

A systematic collection of magnified images from 1256 tooth marks was used with four different DL architectures to generate, via transfer learning, an ensemble analysis of agency classification. The study resulted in a range of 88–80% of accuracy in the classification of the carnivore responsible for each modification depending on the model. This increases by more than 50% the accurate estimates obtained by previously published computer vision analyses of tooth marks. It also opens the possibility to objectively identify specific carnivore agencies in the fossil record. This methodological approach complements other methods, like traditional geometric morphometrics, where discrimination has also been successful. The basal study provides the foundation for future experimental work, in which the reference library should be broadened to provide an even more solid background to any computer-derived discrimination of carnivore agency in taphonomic studies. This will also enable testing a new set of questions that have immense potential and interest for human evolutionary studies.

### Supplementary Information

Supplementary Information.

## Data Availability

Data and code are available at Harvard’s Dataverse public repository: 10.7910/DVN/MLDCIC.
