# Supplementary material for: Computer vision enables taxon-specific identification of African carnivore tooth marks on bone
Source: Sci Rep. 2024 Mar 22;14:6881. doi: 10.1038/s41598-024-57015-z (PMC10959944; doi:10.1038/s41598-024-57015-z)
Supplement: Supplementary file 1 — Supplementary Information. [file 41598_2024_57015_MOESM1_ESM.pdf]

Supplementary Information

Computer vision enables taxon-specific identification of African carnivore tooth marks on bone

Manuel Domínguez-Rodrigo[1,2,3], Marcos Pizarro-Monzo[1,4,5], Gabriel Cifuentes-Alcobendas[1,2], Marina Vegara-Riquelme[1,2], Blanca Jiménez-García[1,2], Enrique Baquedano[1,6],

[1]Institute of Evolution in Africa (IDEA), Alcalá University, Covarrubias 36, 28010 Madrid, Spain.
[2]Area of Prehistory (Department History and Philosophy), University of Alcalá, 28801 Alcalá de Henares, Spain.
[3]Department of Anthropology, Rice University, 6100 Main St., Houston, TX 77005-1827, USA.
[4]Institut Català de Paleoecologia Humana i Evolució Social (IPHES-CERCA), Zona Educacional 4, Campus Sescelades URV (Edifici W3), 43007 Tarragona,Spain.
[5]Universitat Rovira i Virgili (URV), Departament d'Història i Història de l'Art, Avinguda de Catalunya 35, 43002 Tarragona, Spain
[6]Regional Paleontological and Archaeological Museum of Madrid, Plaza de las Bernardas s/n, Alcalá de Henares, Spain.

Models with results

RESNET 50 (SGD, relu)

val_loss: 0.4078 - val_accuracy: 0.8800

Confusion Matrix

```
[[  9   3   6   2]
 [  0  80   6   5]
 [  0   7 126   3]
 [  1   1   5  59]]
```

Classification Report

	precision	recall	f1-score	support
croc	0.90	0.45	0.60	20
hyena	0.88	0.88	0.88	91
leopard	0.88	0.93	0.90	136
lion	0.86	0.89	0.87	66
accuracy			0.88	313
macro avg	0.88	0.79	0.81	313
weighted avg	0.88	0.88	0.87	313

VGG19 (Adagrad,relu)

val_loss: 0.5406 - val_accuracy: 0.8033

Confusion Matrix

```
[[  9   3   5   3]
 [  1  73  16   1]
 [  0  13 122   1]
 [  1   5  15  45]]
```

Classification Report

	precision	recall	f1-score	support
croc	0.82	0.45	0.58	20
hyena	0.78	0.80	0.79	91
leopard	0.77	0.90	0.83	136
lion	0.90	0.68	0.78	66
accuracy			0.80	313
macro avg	0.82	0.71	0.74	313
weighted avg	0.80	0.80	0.79	313

EfficientNetB7 (Adagrad,swish)

val_loss: 0.5012 - val_accuracy: 0.8000

Confusion Matrix

```
[[  6  10   4   0]
 [  0  79   9   3]
 [  2  15 117   2]
 [  1  14   7  44]]
```

Classification Report

	precision	recall	f1-score	support
croc	0.67	0.30	0.41	20
hyena	0.67	0.87	0.76	91
leopard	0.85	0.86	0.86	136
lion	0.90	0.67	0.77	66
accuracy			0.79	313
macro avg	0.77	0.67	0.70	313
weighted avg	0.80	0.79	0.78	313

Densenet (SGD,relu)

val_loss: 0.4095 - val_accuracy: 0.8433

Confusion Matrix

```
[[ 10   0   2   8]
 [  2  72   9   8]
 [  1   7 122   6]
 [  1   2   4  59]]
```

Classification Report

	precision	recall	f1-score	support
croc	0.71	0.50	0.59	20
hyena	0.89	0.79	0.84	91
leopard	0.89	0.90	0.89	136
lion	0.73	0.89	0.80	66
accuracy			0.84	313
macro avg	0.81	0.77	0.78	313
weighted avg	0.84	0.84	0.84	313
